# AMPK regulates lipid accumulation in skeletal muscle cells through FTO-dependent demethylation of *N*^*6*^-methyladenosine

**DOI:** 10.1038/srep41606

**Published:** 2017-02-08

**Authors:** Weiche Wu, Jie Feng, Denghu Jiang, Xihong Zhou, Qin Jiang, Min Cai, Xinxia Wang, Tizhong Shan, Yizhen Wang

**Affiliations:** 1 College of Animal Science, Zhejiang University, Key Laboratory of Animal Nutrition & Feed Sciences, Ministry of Agriculture; Zhejiang Provincial Laboratory of Feed and Animal Nutrition, No. 866 Yuhangtang Road, Hangzhou, Zhejiang, 310058, P. R. China; 2 Institute of Subtropical Agriculture, The Chinese Academy of Science, Changsha, Hunan 410125, P. R. China

## Abstract

Skeletal muscle plays important roles in whole-body energy homeostasis. Excessive skeletal muscle lipid accumulation is associated with some metabolic diseases such as obesity and Type 2 Diabetes. The energy sensor AMPK (AMP-activated protein kinase) is a key regulator of skeletal muscle lipid metabolism, but the precise regulatory mechanism remains to be elucidated. Here, we provide a novel mechanism by which AMPK regulates skeletal muscle lipid accumulation through fat mass and obesity-associated protein (FTO)-dependent demethylation of *N*^*6*^-methyladenosine (m^6^A). We confirmed an inverse correlation between AMPK and skeletal muscle lipid content. Moreover, inhibition of AMPK enhanced lipid accumulation, while activation of AMPK reduced lipid accumulation in skeletal muscle cells. Notably, we found that mRNA m^6^A methylation levels were inversely correlated with lipid content in skeletal muscle. Furthermore, AMPK positively regulated the m^6^A methylation levels of mRNA, which could negatively regulate lipid accumulation in C2C12. At the molecular level, we demonstrated that AMPK regulated lipid accumulation in skeletal muscle cells by regulating FTO expression and FTO-dependent demethylation of m^6^A. Together, these results provide a novel regulatory mechanism of AMPK on lipid metabolism in skeletal muscle cells and suggest the possibility of controlling skeletal muscle lipid deposition by targeting AMPK or using m^6^A related drugs.

The incidence of obesity and Type 2 Diabetes (T2D) is increasing worldwide^1^,^2^, so a better understanding of how ectopic fat deposition occurs remains urgent. Skeletal muscle plays essential roles in whole body energy homeostasis^3^ and insulin action^4^. Notably, excessive lipid accumulation in skeletal muscle is associated with the development of obesity and T2D^5^,^6^. Therefore, studying the underlying mechanism of skeletal muscle lipid metabolism may provide useful information for the treatment of obesity and T2D.

Lipid accumulation and energy metabolism in skeletal muscle are regulated by multiple factors. Genetically, obese mice (known as *ob*/*ob* and *db*/*db* mice) are observed to have greater skeletal muscle lipid^7^. High-fat diet feeding results in greater lipid accumulation in skeletal muscle^8^. In addition, slow myofibres have more intramuscular triglycerides (abbreviated as IMTG) than fast myofibres^9^, indicating that myofibre type is associated with IMTG accumulation. At the molecular level, substantial studies have revealed that several genes have considerable impacts on skeletal muscle lipid metabolism, including ATGL (adipose triglyceride lipase)^10^,^11^,^12^, HSL (hormone-sensitive lipase)^13^ and AMPK^14^,^15^,^16^,^17^,^18^,^19^. AMPK is recognized as a key energy sensor that can regulate energy status^20^. Activation of AMPK increases the level of glucose uptake in skeletal muscle^16^,^17^, enhances lipid oxidation and reduces fatty acid incorporation into triacylglycerol through phosphorylation of ACC2 (acetyl-CoA carboxylase 2) in skeletal muscle^14^,^18^. By contrast, ablation of AMPK reduces fatty acid oxidation and enhances skeletal muscle lipid accumulation and leads to elevated triglyceride content^15^. Furthermore, AMPK is able to facilitate skeletal muscle mitochondrial biogenesis and thus regulates the energy status of skeletal muscle cells^19^. However, more studies are needed to understand the regulatory mechanism of AMPK on skeletal muscle lipid accumulation.

*N*^*6*^-methyladenosine (m^6^A), the most prevalent mRNA modification^21^, is involved in diverse biological processes including lipid accumulation and energy metabolism^22^,^23^. The m^6^A modification can be dynamically modulated by several genes, including the methyltransferases (METTL3, METTL14 and WTAP)^24^,^25^ and the demethylases (ALKBH5 and FTO)^26^,^27^. Depletion of FTO (fat mass and obesity-associated protein) results in an increased mRNA m^6^A level, while overexpression of FTO leads to a decreased m^6^A level. A subsequent study shows that FTO partially co-localizes with nuclear speckles (one organelles that are enriched in pre-mRNA splicing factors and located in the interchromatin nuclear space)^28^, implicating m^6^A in nuclear RNA as a physiological substrate of FTO^26^. Moreover, FTO dynamically regulates m^6^A level and plays a critical role in determining splicing and gene expression patterns^23^. It has been demonstrated that FTO is strongly associated with body mass and obesity^29^. Overexpression of FTO in mice increases fat mass and obesity, while loss of FTO protects mice from obesity^30^,^31^, suggesting a prominent role of FTO in body fat metabolism. A recent study provides evidence that FTO regulates 3T3-L1 preadipocyte differentiation by demethylating m^6^A. In addition, knockdown of METTL3 (methyltransferase like 3) promotes 3T3-L1 preadipocyte differentiation, which together links m^6^A methylation to adipogenesis^23^. More recently, we found that inhibition of mRNA m^6^A methylation enhanced adipogenesis, while increasing the mRNA m^6^A methylation level suppressed adipogenesis in porcine adipocytes^22^. Furthermore, we also observed that m^6^A is involved in the metabolic effects of betaine on adipose tissue and liver^32^,^33^. However, the effects of mRNA m^6^A methylation on lipid metabolism in skeletal muscle remain unclear. Furthermore, whether the AMPK pathway could affect FTO and m^6^A methylation is unknown.

Here, we provide a novel mechanism for AMPK in the regulation of skeletal muscle lipid accumulation through FTO-dependent demethylation of mRNA m^6^A. These results provide new insight into the role of AMPK and FTO in skeletal muscle lipid accumulation and implicate the possibility of controlling skeletal muscle lipid deposition using AMPK or m^6^A related drugs.

## Results

### The phosphorylation of AMPKα is inversely correlated with lipid accumulation in skeletal muscle

**Figure 1:**
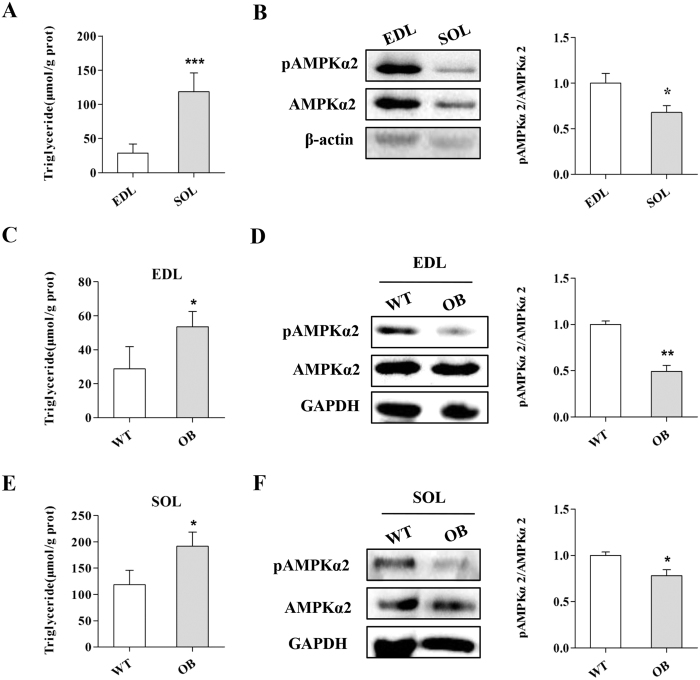
The AMPK activity in skeletal muscle is inversely correlated with lipid content. (**A,B**) The triglyceride content in SOL was much higher than EDL (**A**) while the p-AMPKα2 level in SOL was lower than EDL (**B**). (**C,D**) The EDL of OB mice contained more triglyceride (**C**) but a lower p-AMPKα2 level compared to WT mice (**D**). (**E,F**) The triglyceride content (**E**) and the p-AMPKα2 levels (**F**) in SOL of OB mice (**F**). The results are presented as the mean ± SEM. n = 4–6. *p < 0.05, **p < 0.01, ***p < 0.001.

Slow myofibres contain more IMTG than fast myofibres^9^. To confirm whether AMPK is associated with skeletal muscle lipid accumulation, we first measured the phosphorylation levels of AMPKα2 (p-AMPKα2), the predominant subunit of AMPK in skeletal muscle^34^, in the SOL (soleus; mostly slow myofibres) and EDL (extensor digitorum longus; mostly fast myofibres) of WT mice. Consistent with previous reports^9^, more TG content in SOL was found compared to EDL (Fig. 1A). Conversely, the p-AMPKα2 levels in SOL were lower than EDL (Fig. 1B), indicating that p-AMPK levels inversely correlate with skeletal muscle lipid content. The correlational analysis confirmed the inverse correlation between p-AMPK levels and lipid content in skeletal muscle (Fig. S1A).

OB mice or HFD fed mice have more lipid accumulation in skeletal muscle tissue^7^,^8^. To further confirm the correlation between AMPK and skeletal muscle lipid accumulation, we detected the p-AMPKα2 levels and TG content in OB mice. Notably, we found higher TG content but lower p-AMPKα2 levels in the skeletal muscle tissues of OB mice compared to WT mice (Fig. 1C–F). In addition, inverse correlations between the p-AMPKα2 levels and TG content were found in the EDL and SOL from OB mice (Fig. S1B and C) and HFD fed mice (Figs S2; S3).These results confirmed that the p-AMPKα level was inversely correlated with lipid content in skeletal muscle, suggesting that AMPK may negatively regulate lipid accumulation in skeletal muscle.

### AMPK negatively regulates lipid accumulation in skeletal muscle cells

**Figure 2:**
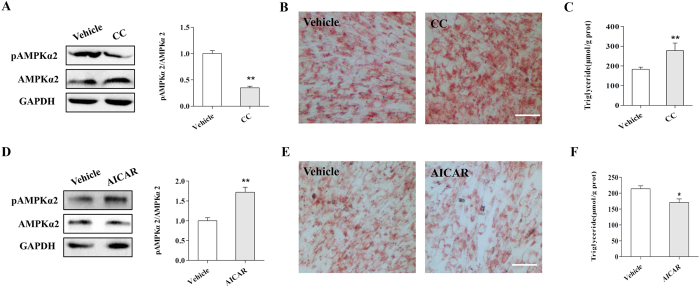
AMPK negatively regulates lipid accumulation in C2C12 cells. (**A**) The p-AMPKα2 level was significantly inhibited by Compound C. (**B,C**) ORO staining (**B**) and triglyceride content (**C**) in C2C12 cells treated withor without Compound C. (**D**) AICAR upregulates p-AMPKα2 expression in C2C12 cells. (**E,F**) ORO staining (**E**) and triglyceride content (**F**) in C2C12 cells treated withor without AICAR. Data are presented as the mean ± SEM. n = 3. *p < 0.05, **p < 0.01.

To directly address the effects of AMPK on lipid metabolism in skeletal muscle cells, we treated adipogenic-differentiated C2C12 cells with an AMPK inhibitor (Compound C) or activator (AICAR). We found that inhibition of AMPK with Compound C significantly decreased the level of p-AMPKα2 (Fig. 2A). In addition, ORO staining and TG measurement results showed that Compound C treatment dramatically elevated lipid accumulation in skeletal muscle cells (Fig. 2B,C). In contrast, activation of AMPK by AICAR upregulated the p-AMPKα2 level (Fig. 2D) but decreased lipid accumulation and TG content in C2C12 cells (Fig. 2E,F). Furthermore, inverse correlations were observed between the p-AMPKα2 levels and triglycerides in the CC and AICAR treatments (Fig. S4). Together, our results demonstrated that AMPK negatively regulated lipid accumulation in skeletal muscle cells.

### mRNA m^6^A methylation inversely affects skeletal muscle lipid accumulation

**Figure 3:**
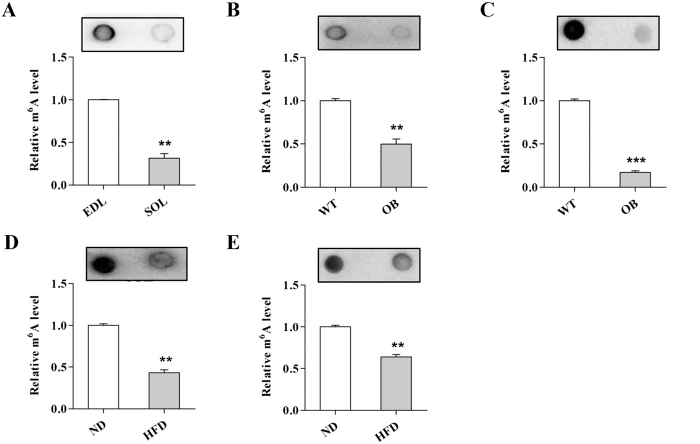
The m^6^A methylation level is inversely correlated with skeletal muscle lipid content. (**A**) The m^6^A level in the EDL and SOL. (**B,C**) The m^6^A level in the EDL (**B**) and SOL (**C**) of WT and OB mice. (**D,E**) The m^6^A level in the EDL (**D**) and SOL (**E**) of the ND and HFD group. The results are presented as the mean ± SEM. n = 4–6. **p < 0.01, ***p < 0.001.

Recent studies provide evidence that mRNA m^6^A methylation regulates adipogenesis in adipocytes^22^,^23^. To examine whether m^6^A methylation affects skeletal muscle lipid accumulation, we first assessed m^6^A levels in slow SOL and fast EDL muscles. Notably, we found that the m^6^A levels in the SOL were much lower compared to the EDL of WT mice (Fig. 3A). Reduced m^6^A levels in the SOL (Fig. 3B) and EDL (Fig. 3C) of OB mice were observed. In addition, compared to the ND group, the SOL (Fig. 3D) and EDL (Fig. 3E) of the HFD group had lower m^6^A levels. These results indicated that mRNA m^6^A methylation might inversely regulate lipid accumulation in skeletal muscle cells.

**Figure 4:**
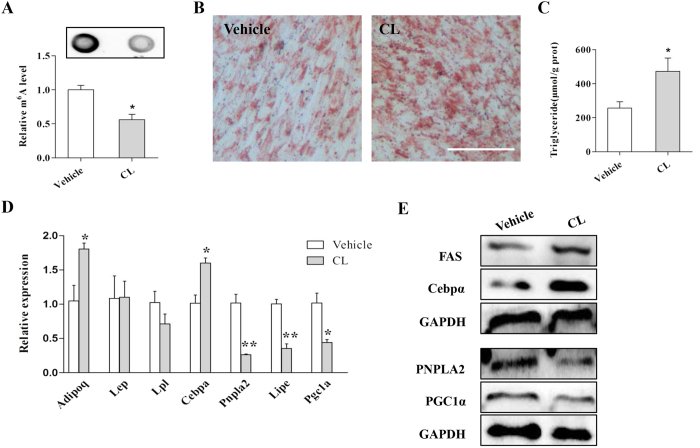
Inhibition of m^6^A methylationin C2C12 cells reduces cellular lipid accumulation. (**A**) Cycloleucine treatment decreased the m^6^A level in C2C12 cells. (**B**) ORO staining of C2C12 cells treated with vehicle or cycloleucine. (**C**) Cycloleucine treatment increased cellular triglyceride content. (**D,E**) Cycloleucine treatment increased the mRNA levels of *Cebpα* but decreased the expression of *pnpla2, Lipe*, and *Pgc1α* (**D**), as well as the protein levels (**E**). CL, cycloleucine. The results are presented as the mean ± SEM. n = 3. *p < 0.05, ** *p* < 0.01. Scale bars: 100 μm.

To address whether mRNA m^6^A methylation modulates lipid metabolism in skeletal muscle cells, we treated C2C12 cells with the methylation inhibitor cycloleucine. The mRNA m^6^A level was significantly decreased by cycloleucine treatment (Fig. 4A). The ORO staining and TG detection results showed much greater TG accumulation following cycloleucine treatment (Fig. 4B,C). Furthermore, real-time PCR analysis revealed that cycloleucine treatment upregulated the expression of the adipogenic gene *Cebpα (CCAAT/enhancer-binding protein alpha*) and decreased the expression of both lipolysis (*Pnpla2* and *Lipe*) and mitochondrial genes (*Pgc1α*) (Fig. 4D). Western blot results further confirmed that inhibition of m^6^A methylation dramatically increased the protein levels of FAS (fatty acid synthase) and C/EBPα but decreased the protein levels of PNPLA2 and PGC1α (peroxisome proliferator activated receptor gamma coactivator 1 alpha) (Fig. 4E). These results indicated that mRNA m^6^A methylation increased lipid accumulation in skeletal muscle cells by affecting the expression of lipid metabolism related genes.

### AMPK decreases lipid accumulation in skeletal muscle cells through upregulation of mRNA m^6^A methylation

**Figure 5:**
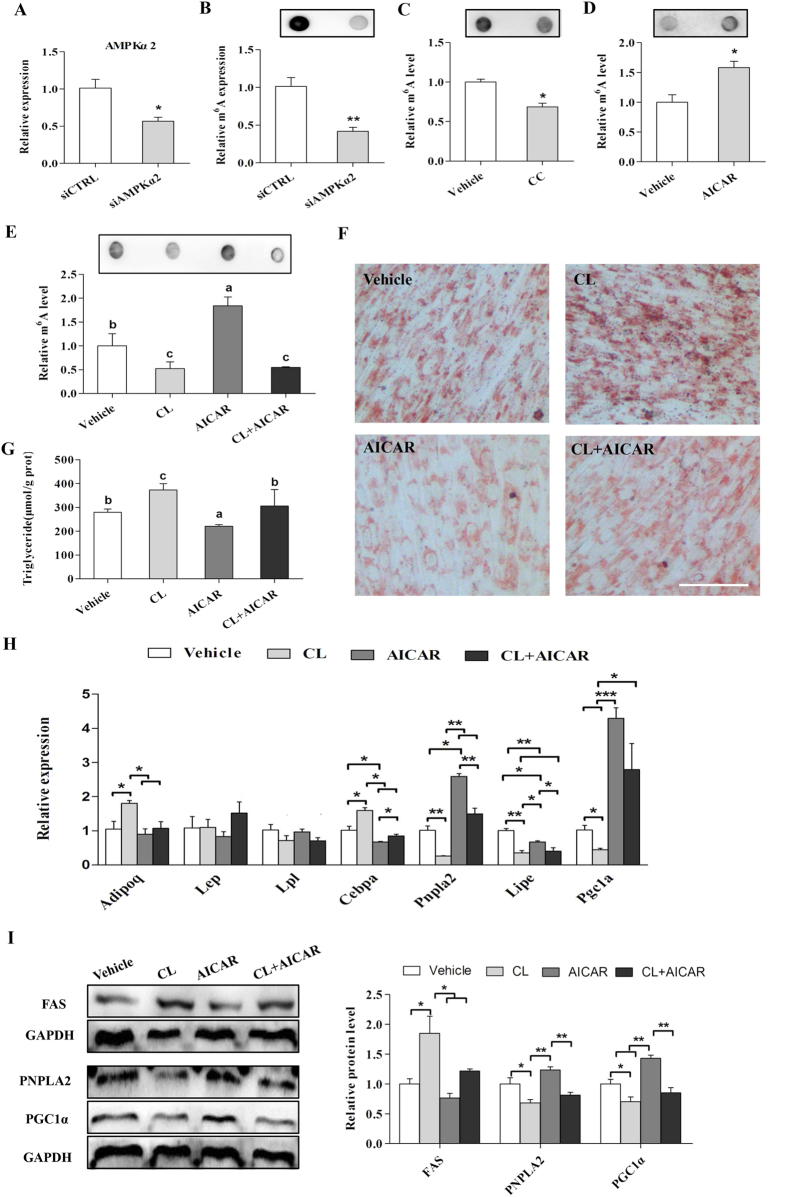
AMPK affects cellular lipid accumulation by regulating m^6^A methylation in C2C12. (**A**) The mRNA levels of AMPKα2 in C2C12 cells treated with AMPKα2 siRNA (siAMPKα2). (**B–D**) m^6^A methylation in C2C12 cells treated with siAMPKα2 (**B**), CC (**C**) or AICAR (**D**). (**E**) The m^6^A levelsin C2C12 cells treated with CL, AICAR or their combination. (**F,G**) ORO staining (**F**) and TG content (**G**) in C2C12 cells treated with CL, AICAR or their combination. (**H,I**) The mRNA and protein levels of the adipogenic and fatty acid lipolysis related genesin C2C12 cells treated with CL, AICAR or their combination. CC, Compound C; CL, cycloleucine. The results are presented as the mean ± SEM. n = 3. *p < 0.05, **p < 0.01, ***p < 0.001. Scale bars: 100 μm.

Next, we determined the effects of AMPK on mRNA m^6^A methylation in skeletal muscle, as our previous study revealed the involvement of AMPK in the m^6^A methylation process in adipocytes^33^. Interestingly, we found that knockdown of AMPKα2 reduced the mRNA m^6^A level in C2C12 cells (Fig. 5A and B). A similar result was observed following Compound C treatment (Fig. 5C).By contrast, AICAR (an AMPK activator) treatment increased the mRNA m^6^A methylation level in C2C12 cells (Fig. 5D). These results combined with our previous reports suggest that AMPK upregulates m^6^A methylation in skeletal muscle cells.

To further examine whether inhibition of mRNA m^6^A methylation can rescue the effect of AMPK activation on reducing lipid accumulation in skeletal muscle cells, we treated adipogenic differentiated C2C12 cells with cycloleucine in combination with AICAR. We found that cycloleucine reduced the m^6^A methylation level while AICAR increased the m^6^A level, but the combined treatment of cycloleucine and AICAR led to a compromised mRNA m^6^A level similar to the cycloleucine group (Fig. 5E). Notably, cycloleucine rescued the lipid accumulation that was downregulated by AICAR (Fig. 5F and G). Additionally, cycloleucine treatment upregulated the *Cebp*α expression that was decreased by AICAR (Fig. 5H). The expression of *Pnpla2* and *Pgc1α* was much higher in the AICAR groups but was decreased by cycloleucine treatment (Fig. 5H). Consistently, Western blot results further confirmed that inhibition of mRNA m^6^A methylation with cycloleucine alleviated the effects of AICAR on the protein levels of FAS, PNPLA2 and PGC1α in C2C12 cells (Fig. 5I). These results showed that AMPK decreased lipid accumulation in skeletal muscle cells by upregulating mRNA m^6^A methylation.

### FTO is essential for reduced m^6^A methylation and increased lipid accumulation in skeletal muscle cells

**Figure 6:**
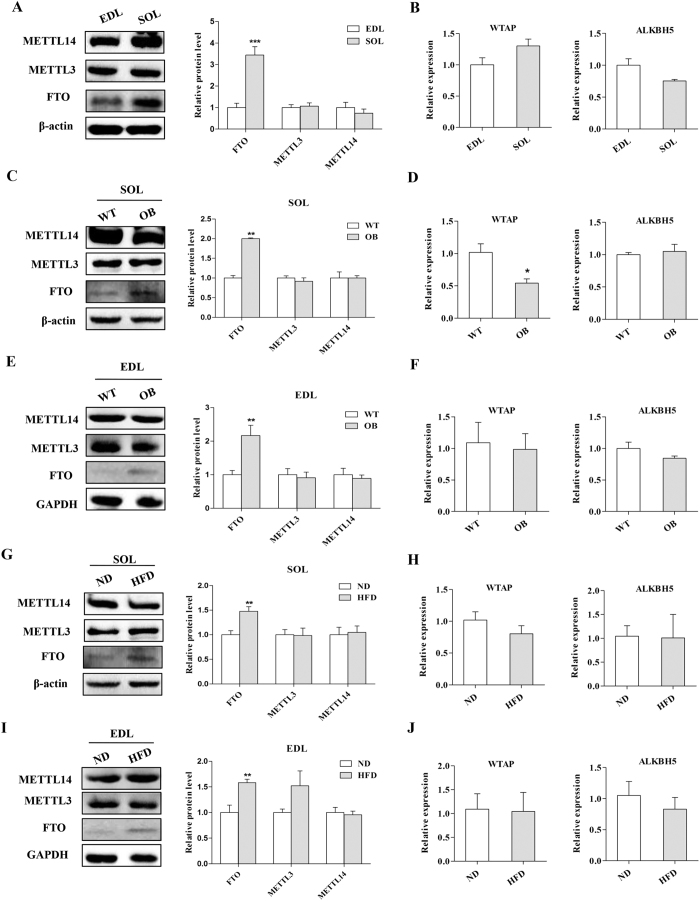
The expression of FTO is upregulated in the skeletal muscle of OB mice and HFD group mice. (**A,B**) The protein levels of FTO, METTL3 and METTL14 (**A**) and the relative mRNA levels of *WTAP* and *ALKBH5* (**B**) in the EDL and SOL of WT mice. (**C,D**) The protein expression of FTO, METTL3 and METTL14 (**C**) and the mRNA levels of *WTAP* and *ALKBH5* (**D**) in the SOL of WT and OB mice. (**E,F**) The protein levels of FTO, METTL3 and METTL14 (**E**) and the mRNA levels of *WTAP* and *ALKBH5* (**F**) in the EDL of WT and OB mice. (**G,H**) The protein expression levels of FTO, METTL3 and METTL14 (**G**) and the relative expression of *WTAP* and *ALKBH5* (**H**) in the SOL of the ND and HFD group. (**I,J**) The protein expression of FTO, METTL3 and METTL14 (**I**) and the relative expression of *WTAP* and *ALKBH5* (**J**) in the EDL of the ND and HFD groups. The results are presented as the mean ± SEM. n = 4–6. *p < 0.05, ** *p* < 0.01, ***p < 0.001.

To understand the molecular mechanism by which AMPK regulates m^6^A methylation and lipid accumulation in skeletal muscle cells, we investigated the expression of m^6^A methylation related methyltransferases (METTL3, METTL14 and WTAP)^24^,^25^ and demethylases (FTO and ALKBH5)^26^,^27^. We found that the protein expression levels of METTL3 and METTL14 (methyltransferase like 14) as well as the relative gene expression levels of *WTAP (Wilms tumor 1 associated protein*) and *ALKBH5 (alkB homolog 5*) were similar between SOL and EDL (Fig. 6A and B). However, the protein levels of FTO were significantly higher in the SOL compared to EDL (Fig. 6A). Moreover, we found that the expression of FTO in the SOL of OB mice was upregulated, but the expression of the other m^6^A methylation and demethylation related genes was not obviously changed or was even reduced (Fig. 6C and D). Likewise, FTO expression was upregulated in the EDL of OB mice compared to WT mice (Fig. 6E and F). Consistently, a higher expression of FTO was found in the skeletal muscles of the HFD group compared to the ND group (Fig. 6G and H). However, the expression of the other m^6^A methyltransferases and demethylation genes in skeletal muscle tissues was similar between the HFD group and ND group (Fig. 6I and J). Taken together, our data demonstrated that the mRNA m^6^A methylation levels in skeletal muscle might be negatively regulated by FTO.

**Figure 7:**
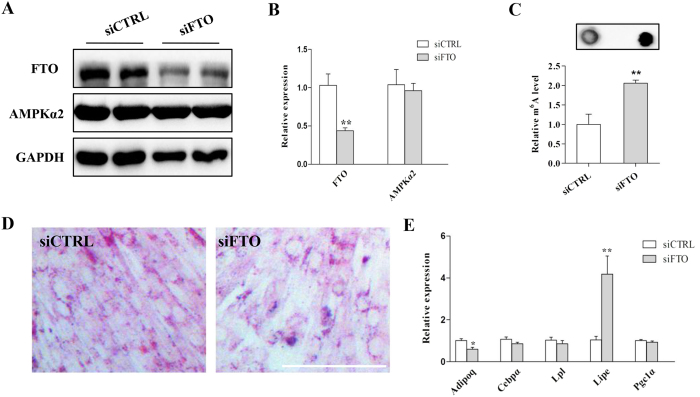
FTO is essential for m^6^A methylation and lipid accumulation in skeletal muscle cells. (**A,B**) The protein (**A**) and mRNA (**B**) levels of FTO in C2C12 cells after knockdown of FTO with siRNA (siFTO). (**C**) The level of mRNA m^6^A methylation was increased after FTO knockdown. (**D**) ORO staining of C2C12 cells after knockdown of FTO. (**E**) The expression of lipid metabolism related genes after FTO knockdown. The results are presented as the mean ± SEM. n = 3–5. *p < 0.05, ** *p* < 0.01. Scale bars: 100 μm.

We next examined the effect of FTO on adipogenic-differentiation in C2C12 cells. The mRNA and protein expression of FTO were significantly reduced by siRNA (Fig. 7A and B). The mRNA m^6^A methylation level was increased by knockdown of FTO with siRNA (Fig. 7C). ORO staining results indicated that the cellular lipid content was decreased in the FTO knockdown group compared to the control group (Fig. 7D). Real-time PCR analysis showed that *Adipoq* expression was inhibited while *Lipe* expression was upregulated when FTO was knocked down (Fig. 7E). Notably, we found that knockdown of FTO did not affect the expression of AMPKα2 (Fig. 7A and B). These results revealed that FTO was essential for maintaining a normal m^6^A methylation level and lipid accumulation in skeletal muscle cells.

### AMPK negatively regulates mRNA m^6^A methylation through FTO-dependent demethylation

**Figure 8:**
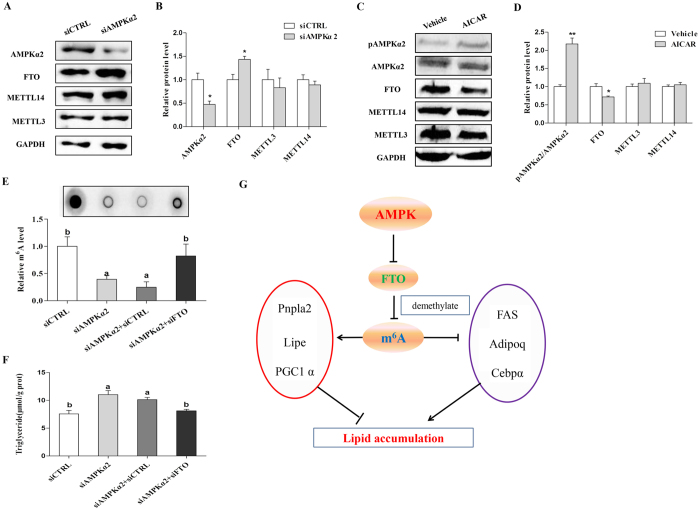
AMPK regulates lipid accumulation and m^6^A methylation through FTO-dependent demethylation in C2C12 cells. (**A,B**) The protein levels of AMPKα2, FTO, METTL3 and METTL14 in C2C12 cells after knockdown of AMPKα2. The relative expression levels of AMPKα2, METTL3, METTL14 and FTO were normalized to GAPDH. (**C,D**) The protein levels of AMPKα2, p-AMPKα2, FTO, METTL3 and METTL14 in C2C12 cells after AICAR treatment. The relative protein levels of p-AMPKα2, METTL3, METTL14 and FTO were normalized to GAPDH in C2C12 cells after AICAR treatment. (**E**) The m^6^A methylation levels after treatment with siAMPKα2, siFTO or their combination. (**F**) The triglyceride content after treatment with siAMPKα2, siFTO or their combination. (**G**) A model depicting the role of AMPK in the regulation of lipid accumulation and m^6^A methylation through FTO-dependent demethylation in skeletal muscle cells. CC, Compound C. Data are presented as the mean ± SEM. n = 3. *p < 0.05, **p < 0.01.

To directly determine whether AMPK negatively regulates skeletal muscle mRNA m^6^A methylation and lipid metabolism through FTO, we treated C2C12 cells with Compound C. Western blot analysis revealed that the p-AMPKα2 level was reduced while the expression of FTO was increased after Compound C treatment (Fig. S5A and B). Furthermore, when AMPKα2 was knocked down by siRNA, FTO expression was enhanced in C2C12 cells (Fig. 8A and B). By contrast, activation of AMPK by AICAR suppressed FTO protein expression (Fig. 8C and D). We next performed co-transfection of AMPKα2 and FTO in C2C12 cells. When AMPKα2 was knocked down, the m^6^A methylation level was reduced. However, the m^6^A methylation level was restored when C2C12 cells were transfected with both siAMPKα2 and siFTO (Fig. 8E). Moreover, the triglyceride content was also restored when both siAMPKα2 and siFTO were transfected into C2C12 cells (Fig. 8F). Similar results were also observed when C2C12 cells were treated with Compound C and siFTO (Fig. S5C and D). Based on our above findings, we conclude that AMPK inversely regulates FTO expression and subsequently affected m^6^A methylation and cellular lipid accumulation in skeletal muscle cells (Fig. 8G).

## Discussion

In this study, we provide a novel mechanism for AMPK in the regulation of skeletal muscle lipid accumulation through FTO-dependent demethylation of m^6^A. We first confirmed the inverse correlation between AMPK and skeletal muscle lipid content and subsequently revealed the downregulation of AMPK on lipid accumulation in skeletal muscle cells. Interestingly, we found that the m^6^A methylation levels of mRNA were inversely correlated with lipid content in skeletal muscle cells. Furthermore, AMPK decreases lipid accumulation in skeletal muscle cells by regulating mRNA m^6^A methylation. At the molecular level, we demonstrated that AMPK decreases mRNA m^6^A methylation and lipid accumulation in skeletal muscle cells through FTO-dependent demethylation. Our study provides new insight into the controlling of lipid accumulation in skeletal muscle cells and the treatment of obesity or T2D by downregulating AMPK or mRNA m^6^A methylation.

Intracellular triglycerides are functionally important in health and disease^35^. Intramuscular triglycerides have long been shown to be associated with metabolic dysfunction, including insulin resistance, obesity and T2D^4^,^5^,^6^. However, recent studies have increasingly challenged this accepted paradigm^36^. For instance, although IMTG content and insulin sensitivity have a strong negative correlation^37^, emerging evidence argues that the development of obesity and insulin resistance is due to increased fat oxidation^36^. Based on these findings, accretion of TG is an association with skeletal muscle insulin resistance and T2D, but is not known to be causative. Furthermore, studies have found that MCK-PGC1α mice have increased mitochondrial content accompanied with increased IMTG accumulation, which indicates that mitochondrial function may not be related to IMTG accumulation^38^. Moreover, elevated IMTG content is observed when the transcriptional regulator of skeletal muscle glucose utilization genes, Nur77^39^, is ablated, suggesting that glucose utilization may not be involved in the IMTG accumulation process. Because of this, it is urgent to better understand the underlying mechanism of IMTG metabolism, and our study contributes to understanding the accumulation pattern of IMTG.

AMPK is the major cellular energy sensor^20^ and acts as a critical mediator in skeletal muscle lipid metabolism^14^,^15^,^16^,^17^,^18^,^19^. Here, we confirmed the inverse correlation between AMPK and skeletal muscle lipid content. Higher expressions of adipogenic genes but lower expressions of AMPKα2 were observed in SOL muscle compared to EDL muscle^40^. Furthermore, enhanced lipid content was observed in the skeletal muscle from obese subjects^9^. Previous studies found that AMPK activity is downregulated in obese mice skeletal muscle^41^ and that low AMPK activity may be related to the abnormal status of muscle fatty acid metabolism in obesity^42^. Likewise, HFD feeding increases lipid content but decreases the p-AMPK level in skeletal muscle^43^,^44^. Our results from the skeletal muscles of OB mice and HFD fed mice were in accordance with these reports. In addition, we also found that AMPK inhibition enhanced cellular lipid content while AMPK activation reduced lipid content in C2C12 cells. Our results are consistent with previous reports^45^,^46^ and demonstrate that AMPK acts as a negative regulator of lipid accumulation in skeletal muscle cells.

Previous reports have shown that AMPK regulates lipid accumulation by activating ATGL to increase lipolysis^47^ and decreases C/EBPα and FAS expression to inhibit fat synthases^40^. Here, we found that AMPK negatively regulated lipid accumulation in skeletal muscle cells by regulating mRNA m^6^A methylation. It has been reported that mRNA m^6^A methylation is involved in lipid metabolism and fat synthesis in different tissues including the liver^48^ and adipose tissue^33^. In 3T3-L1 cells, increasing the mRNA m^6^A methylation level inhibits differentiation while decreasing the mRNA m^6^A methylation level promotes 3T3-L1 differentiation, indicating that mRNA m^6^A modification plays a crucial role during adipogenesis^23^. Recently, our previous study showed that inhibition of mRNA m^6^A methylation by cycloleucine enhanced adipogenesis while increasing mRNA m^6^A methylation level via the methyl donor betaine suppressed adipogenesis in porcine adipocytes^22^. We found that mRNA m^6^A methylation inversely modulated lipid accumulation in skeletal muscle cells. Inhibition of m^6^A methylation decreased the expression of ATGL, HSL and PGC1α while increasing the expression of C/EBPα in C2C12 cells. ATGL and HSL are the two key enzymes in lipolysis^11^,^13^. PGC1α promotes mitochondrial biogenesis and thus facilitates fat oxidation in skeletal muscle^49^. C/EBPα belongs to the CCAAT/enhancer-binding protein family and is essential for lipid accumulation^50^. mRNA m^6^A methylation upregulates skeletal muscle lipid accumulation, likely through upregulation of lipid synthase related genes (C/EBPα and FAS) and downregulation of lipolysis and oxidation related genes (ATGL, HSL, PGC1α). mRNA m^6^A methylation is involved in adipogenesis^23^ and may be regulated by AMPK^33^. We found that AMPK positively regulated the m^6^A level in C2C12 cells. Inhibition of mRNA m^6^A methylation could alleviate the effect of AMPK on lipid accumulation in skeletal muscle cells. Together, we demonstrated that AMPK negatively affects lipid accumulation in skeletal muscle cells, at least partially, by mediating m^6^A methylation.

mRNA m^6^A methylation is controlled by methyltransferases (METTL3, METTL14 and WTAP)^24^,^25^ and demethylases (FTO and ALKBH5)^26^,^27^. We demonstrate that AMPK regulates lipid accumulation and mRNA m^6^A methylation through FTO dependent demethylation. The FTO gene is widely expressed in peripheral regions related to energy homeostasis, including the liver, adipose tissue and skeletal muscle^51^. FTO plays an important role in demethylation. A recent study revealed that FTO could efficiently demethylate m^6^A in nuclear RNA by co-localizing with nuclear speckles^26^. Previous studies demonstrated that FTO may be crucial for normal skeletal muscle lipid accumulation and function^52^,^53^,^54^. The effect of FTO on adipocyte proliferation and differentiation requires its demethylase activity^23^,^55^. Our data showed that the expression of FTO was upregulated in the skeletal muscle of HFD and OB mice and therefore demethylated their methylated mRNA m^6^A. Moreover, our data revealed that inhibition of AMPK upregulated FTO expression while activation of AMPK downregulated FTO expression, which, to our knowledge, is the first instance that the relationship between AMPK and FTO in C2C12 cells has been elucidated. Consistently, our previous study showed that AMPK inversely regulates FTO in adipose tissues^33^. Other studies also showed that AMPK may inversely regulate FTO expression^56^,^57^. Nevertheless, further studies are needed to elucidate the exact mechanism by which AMPK regulates FTO and m^6^A methylation in skeletal muscle cells.

In summary, we demonstrate that AMPK plays a critical role in regulating m^6^A levels and lipid accumulation in skeletal muscle cells. Our study revealed that FTO-dependent demethylation of mRNA m^6^A methylation was involved in the regulation of skeletal muscle lipid accumulation by the AMPK signalling pathway. These results enhance our understanding of the role of AMPK in the regulation of lipid accumulation and mRNA m^6^A methylation in skeletal muscle cells and provide new insights into the molecular regulation of lipid metabolism and metabolic diseases.

## Materials and Methods

### Animals and experimental protocol

All of the animal procedures were approved by the Committee of Experimental Animal Care of Zhejiang University (Hangzhou, China). All of the experimental methods were performed in accordance with approved guidelines.

Sixteen 10-wk-old male C57BL/6 J mice [wild-type (WT) mice] and six 10-wk-old male obese C57BL/6J^*ob*/*ob*^ (OB) mice were purchased from Nanjing Biomedical Research Institute of Nanjing University. WT mice were randomly divided into 2 groups (8 mice/group): normal chow diet (ND) and high-fat diet (HFD) groups. The ND group was fed with a normal chow diet containing10% fat, 20% protein and 70% carbohydrate. The HFD group was fed a HFD containing 45% fat, 20% protein and 35% carbohydrate. OB mice were fed with ND. All mice were housed at 22 ± 1 °C under a 12-h light cycle with free access to water and diet during the experiment. After 8 weeks, the extensor digitorumlongus (EDL) and soleus (SOL) muscles were collected for triglyceride detection or were immediately snap-frozen in liquid nitrogen and stored at −80 °C for total RNA and protein extraction.

### Cell culture and adipogenic differentiation

The mouse myoblast cell line C2C12 was maintained in DMEM containing 10% fetal bovine serum (FBS) in 5% CO_2_ at 37 °C. For adipogenic differentiation, the cells were induced with adipogenic induction medium containing 0.5 μM 3-isobuty-1-methylxanthine (IBMX), 1 μM dexamethasone (DEXA) and 167 nM insulin for 2 days and then induced in differentiation medium containing 167 nM insulin for 2 days, followed by culturing with 10% FBS/DMEM medium until analysis.

### Cell treatment

Cell treatment was performed after adipogenic differentiation. To activate AMPK, the cells were treated with 1 mM AICAR (Selleckchem, Houston, USA) for 24 hours. To inhibit AMPK, the cells were treated with 10 μM Compound C (Selleckchem, Houston, USA) for 24 hours. To inhibit m^6^A methylation, the cells were treated with 10 μM cycloleucine (Sigma, USA) for 24 hours.

### siRNA transfection

siRNA against FTO or AMPKα2 and a negative control (siCTRL) were designed and produced by RiboBio Company (Guangzhou, China). Twenty-four hours before transfection, cells were plated without any antibiotics. Then, 100 nM specific siRNA or siCTRL was transfected into C2C12 myoblasts using Lipofectamine 2000 (Invitrogen).

### Oil Red O (ORO) staining and triglyceride detection

Cultured cells were washed with PBS and fixed with 10% formaldehyde for 5 min at room temperature. Then, all formaldehyde was removed and ORO working solutions were added containing 6 ml ORO stock solution (5 mg/ml in isopropanol) and 4 ml dH_2_O for 10 min. After staining, cells were washed with dH_2_O and then imaged. The triglyceride (TG) levels in skeletal muscle and cultured C2C12 cells were determined using a triglyceride determination kit (Applygen, China).

### RNA extraction and quantitative RT-PCR

The total RNA from all skeletal muscle and cells was extracted using the TRIzol Reagent (Invitrogen) according to the manufacturer′s instructions. RevertAid Reverse Transcriptase (Fermentas) was used to synthesize cDNA. Real-time PCR was performed with the StepOnePlus^TM^ Real Time System (Applied Biosystems); the reaction contained 4 steps: 50 °C for 2 min, 95 °C for 10 min followed by 40 cycles at 95 °C for 10 s, and then 60 °C for 30 s. All primers used in this study were listed in Table 1. The 2^−ΔΔCt^ method was used to quantify mRNA expression relative to 18s rRNA.

### Western blotting analysis

Proteins from all the skeletal muscles and cells were extracted using the Protein Extraction Reagent (KeyGENBioTECH, Nanjing, China), and the concentrations were determined with the BCA Assay Kit (KeyGENBioTECH, Nanjing, China). Next, equal amounts of protein were separated on 10% SDS-PAGE gels followed by electrotransfer to polyvinylidenedifluoride membranes (PVDF). After blocking with 5% skim milk powder in 0.05% TBST, the membranes were incubated with the following primary antibodies: FTO, phospho-AMPKα2 (Abcam), AMPKα2 (GeneTex), METTL3 (Proteintech), METTL14 (Sigma), FAS, C/ebpα, ATGL, PGC1α (Santa Cruz), GAPDH (Boster) and β-actin (Hua An, Hangzhou, China). The secondary antibodies were HRP-conjugated anti-mouse and anti-rabbitIgG (Hua An, Hangzhou, China). Finally, the immunocomplexes were detected using the ECL Western Blotting Detection System (Amersham, Biosciences, Piscataway, NJ, USA).

### Dot-blot assay

To analyse the level of mRNA m^6^A methylation, a dot blot assay was performed according to a previously reported method^26^. Briefly, isolated RNA was first denatured at 95 °C for 3 min, followed by immediate chilling on ice. The denatured RNA was then spotted on an NC membrane optimized for nucleic acid transfer. After UV cross-linking in a StratageneStratalinker 2400 UV Crosslinker, the membrane was washed with 1× TBST, blocked with 5% of non-fat milk in TBST, and incubated with anti-m^6^A antibody (1:2000, Synaptic Systems) overnight at 4 °C. After incubating with a corresponding peroxidase-conjugated secondary antibody for 1 h at room temperature, the membrane was detected using the ECL Western Blotting Detection System (Amersham, Biosciences, Piscataway, NJ, USA). To ensure an equal amount of RNA was spotted on the membrane, the same blot was stained with 0.02% methylene blue in 0.3 M sodium acetate (pH 5.2).

### Statistical analysis

All data are presented as means ± SEM. Each cell culture experiment was performed in triplicate and that results presented represent the findings from 2–3 individual experiments. We used Pearson correlation analysis to determine correlations. The statistical significance of differences in the mean values between groups was assessed by Student’s t-test, and a *p* value of less than 0.05 was considered statistically significant.

## Additional Information

**How to cite this article**: Wu, W. *et al*. AMPK regulates lipid accumulation in skeletal muscle cells through FTO-dependent demethylation of *N*^6^-methyladenosine. *Sci. Rep.* **7**, 41606; doi: 10.1038/srep41606 (2017).

**Publisher's note:** Springer Nature remains neutral with regard to jurisdictional claims in published maps and institutional affiliations.

## Supplementary Material

Supplemental Data

## Figures and Tables

**Table 1:** Primers used for real-time PCR.

Genes	Primers	Sequences
18S	Forward	TAACCCGTTGAACCCCATT
	Reverse	CCATCCAATCGGTAGTAGCG
Adipoq	Forward	CCGGGACTCTACTACTTCTCTT
	Reverse	TTCCTGATACTGGTCGTAGGT
Alkbh5	Forward	GGTGTTTCTCCTCCCTTTAGTC
	Reverse	CAGAGCAGGCACATCTTCTTA
AMPKα2	Forward	AGACTTAACAGCCTGGAACATAC
	Reverse	CTACGGGCAGTCTCACATTTAG
C/EBPα	Forward	GTAACCTTGTGCCTTGGATACT
	Reverse	GGAAGCAGGAATCCTCCAAATA
FTO	Forward	CAGTTCTGGTTTCAAGGCAATC
	Reverse	TGTCATGCTCTCCATCTTCTTC
Leptin	Forward	GGTTGATCTCACAATGCGTTTC
	Reverse	TGGGAGACAGGGTTCTACTT
Lipe	Forward	CATCAACCACTGTGAGGGTAAG
	Reverse	AAGGGAGGTGAGATGGTAACT
Lpl	Forward	GCCCGAGGTTTCCACAAATA
	Reverse	GCTGAAGTAGGAGTCGCTTATC
Pgc1α	Forward	GGATTGAAGTGGTGTAGCGAC
	Reverse	GCTCATTGTTGTACTGGTTGGA
Pnpla2	Forward	TAGCTAACAGTTGGGCTTCAC
	Reverse	CAGAGAGAACAGAGCAGCTTAC
WTAP	Forward	GTACCAGCAGGACTACATCTTC
	Reverse	CTGCCATTACTTGGTCCATTTG

## References

[b1] Whiting D. R., Guariguata L., Weil C. & Shaw J. IDF diabetes atlas: global estimates of the prevalence of diabetes for 2011 and 2030. Diabetes research and clinical practice 94, 311–321 (2011). 22079683 10.1016/j.diabres.2011.10.029

[b2] Finucane M. M. . National, regional, and global trends in body-mass index since 1980: systematic analysis of health examination surveys and epidemiological studies with 960 country-years and 9·1 million participants. The Lancet 377, 557–567 (2011). 10.1016/S0140-6736(10)62037-5 PMC4472365 21295846

[b3] Defronzo R. A., Ferrannini E., Sato Y. & Felig P. Synergistic Interaction between Exercise and Insulin on Peripheral Glucose-Uptake. J Clin Invest 68, 1468–1474 (1981). 7033285 10.1172/JCI110399 PMC370949

[b4] Pan D. A. . Skeletal muscle triglyceride levels are inversely related to insulin action. Diabetes 46, 983–988 (1997). 9166669 10.2337/diab.46.6.983

[b5] Goodpaster B. H., Theriault R., Watkins S. C. & Kelley D. E. Intramuscular lipid content is increased in obesity and decreased by weight loss. Metabolism 49, 467–472 (2000). 10778870 10.1016/s0026-0495(00)80010-4

[b6] DeFronzo R. A. Pathogenesis of type 2 diabetes mellitus. Med Clin N Am 88, 787-+ (2004). 15308380 10.1016/j.mcna.2004.04.013

[b7] Akhmedov D. & Berdeaux R. The effects of obesity on skeletal muscle regeneration. Frontiers in physiology 4, 371 (2013). 24381559 10.3389/fphys.2013.00371 PMC3865699

[b8] Hegarty B. D., Cooney G. J., Kraegen E. W. & Furler S. M. Increased efficiency of fatty acid uptake contributes to lipid accumulation in skeletal muscle of high fat-fed insulin-resistant rats. Diabetes 51, 1477–1484 (2002). 11978645 10.2337/diabetes.51.5.1477

[b9] He J., Watkins S. & Kelley D. E. Skeletal muscle lipid content and oxidative enzyme activity in relation to muscle fiber type in type 2 diabetes and obesity. Diabetes 50, 817–823 (2001). 11289047 10.2337/diabetes.50.4.817

[b10] Badin P. M. . Altered skeletal muscle lipase expression and activity contribute to insulin resistance in humans. Diabetes 60, 1734–1742 (2011). 21498783 10.2337/db10-1364 PMC3114384

[b11] Haemmerle G. . Defective lipolysis and altered energy metabolism in mice lacking adipose triglyceride lipase. Science 312, 734–737 (2006). 16675698 10.1126/science.1123965

[b12] Nunes P. M. . Increased intramyocellular lipids but unaltered *in vivo* mitochondrial oxidative phosphorylation in skeletal muscle of adipose triglyceride lipase-deficient mice. American journal of physiology. Endocrinology and metabolism 303, E71–81 (2012). 22496349 10.1152/ajpendo.00597.2011

[b13] Haemmerle G. . Hormone-sensitive lipase deficiency in mice causes diglyceride accumulation in adipose tissue, muscle, and testis. J Biol Chem 277, 4806–4815 (2002). 11717312 10.1074/jbc.M110355200

[b14] Collier C. A., Bruce C. R., Smith A. C., Lopaschuk G. & Dyck D. J. Metformin counters the insulin-induced suppression of fatty acid oxidation and stimulation of triacylglycerol storage in rodent skeletal muscle. American journal of physiology. Endocrinology and metabolism 291, E182–189 (2006). 16478780 10.1152/ajpendo.00272.2005

[b15] Fujii N. . Ablation of AMP-activated protein kinase alpha2 activity exacerbates insulin resistance induced by high-fat feeding of mice. Diabetes 57, 2958–2966 (2008). 18728234 10.2337/db07-1187 PMC2570392

[b16] Kurth-Kraczek E. J., Hirshman M. F., Goodyear L. J. & Winder W. W. 5′ AMP-activated protein kinase activation causes GLUT4 translocation in skeletal muscle. Diabetes 48, 1667–1671 (1999). 10426389 10.2337/diabetes.48.8.1667

[b17] Merrill G. F., Kurth E. J., Hardie D. G. & Winder W. W. AICA riboside increases AMP-activated protein kinase, fatty acid oxidation, and glucose uptake in rat muscle. The American journal of physiology 273, E1107–1112 (1997). 9435525 10.1152/ajpendo.1997.273.6.E1107

[b18] O’Neill H. M. . AMPK phosphorylation of ACC2 is required for skeletal muscle fatty acid oxidation and insulin sensitivity in mice. Diabetologia 57, 1693–1702 (2014). 24913514 10.1007/s00125-014-3273-1

[b19] Zong H. H. . AMP kinase is required for mitochondrial biogenesis in skeletal muscle in response to chronic energy deprivation. P Natl Acad Sci USA 99, 15983–15987 (2002). 10.1073/pnas.252625599 PMC138551 12444247

[b20] Hardie D. G., Ross F. A. & Hawley S. A. AMPK: a nutrient and energy sensor that maintains energy homeostasis. Nature reviews. Molecular cell biology 13, 251–262 (2012). 22436748 10.1038/nrm3311 PMC5726489

[b21] Fu Y., Dominissini D., Rechavi G. & He C. Gene expression regulation mediated through reversible m(6)A RNA methylation. Nature reviews. Genetics 15, 293–306 (2014). 10.1038/nrg3724 24662220

[b22] Wang X., Zhu L., Chen J. & Wang Y. mRNA m(6)A methylation downregulates adipogenesis in porcine adipocytes. Biochemical and biophysical research communications 459, 201–207 (2015). 25725156 10.1016/j.bbrc.2015.02.048

[b23] Zhao X. . FTO-dependent demethylation of N6-methyladenosine regulates mRNA splicing and is required for adipogenesis. Cell Res 24, 1403–1419 (2014). 25412662 10.1038/cr.2014.151 PMC4260349

[b24] Liu J. . A METTL3-METTL14 complex mediates mammalian nuclear RNA N6-adenosine methylation. Nat Chem Biol 10, 93–95 (2014). 24316715 10.1038/nchembio.1432 PMC3911877

[b25] Ping X. L. . Mammalian WTAP is a regulatory subunit of the RNA N6-methyladenosine methyltransferase. Cell Res 24, 177–189 (2014). 24407421 10.1038/cr.2014.3 PMC3915904

[b26] Jia G. F. . N6-Methyladenosine in nuclear RNA is a major substrate of the obesity-associated FTO (vol 7, pg 885, 2011). Nat Chem Biol 8, 1008–1008 (2012). 10.1038/nchembio.687 PMC3218240 22002720

[b27] Zheng G. Q. . ALKBH5 Is a Mammalian RNA Demethylase that Impacts RNA Metabolism and Mouse Fertility. Mol Cell 49, 18–29 (2013). 23177736 10.1016/j.molcel.2012.10.015 PMC3646334

[b28] Spector D. L. & Lamond A. I. Nuclear speckles. Cold Spring Harbor perspectives in biology 3 (2011). 10.1101/cshperspect.a000646 PMC3039535 20926517

[b29] Frayling T. M. . A common variant in the FTO gene is associated with body mass index and predisposes to childhood and adult obesity. Science 316, 889–894 (2007). 17434869 10.1126/science.1141634 PMC2646098

[b30] Church C. . Overexpression of Fto leads to increased food intake and results in obesity. Nature genetics 42, 1086–1092 (2010). 21076408 10.1038/ng.713 PMC3018646

[b31] Fischer J. . Inactivation of the Fto gene protects from obesity. Nature 458, 894–898 (2009). 19234441 10.1038/nature07848

[b32] Chen J., Zhou X., Wu W., Wang X. & Wang Y. FTO-dependent function of N6-methyladenosine is involved in the hepatoprotective effects of betaine on adolescent mice. Journal of physiology and biochemistry 71, 405–413 (2015). 26078098 10.1007/s13105-015-0420-1

[b33] Zhou X. . The beneficial effects of betaine on dysfunctional adipose tissue and N6-methyladenosine mRNA methylation requires the AMP-activated protein kinase alpha1 subunit. J Nutr Biochem 26, 1678–1684 (2015). 26365580 10.1016/j.jnutbio.2015.08.014

[b34] Birk J. B. & Wojtaszewski J. F. Predominant alpha2/beta2/gamma3 AMPK activation during exercise in human skeletal muscle. The Journal of physiology 577, 1021–1032 (2006). 17038425 10.1113/jphysiol.2006.120972 PMC1890393

[b35] Meex R. C. R., Schrauwen P. & Hesselink M. K. C. Modulation of myocellular fat stores: lipid droplet dynamics in health and disease. Am J Physiol-Reg I 297, R913–R924 (2009). 10.1152/ajpregu.91053.2008 19692657

[b36] Muoio D. M. Intramuscular triacylglycerol and insulin resistance: Guilty as charged or wrongly accused? Bba-Mol Cell Biol L 1801, 281–288 (2010). 10.1016/j.bbalip.2009.11.007 PMC4428562 19958841

[b37] Goodpaster B. H. & Kelley D. E. Skeletal muscle triglyceride: marker or mediator of obesity-induced insulin resistance in type 2 diabetes mellitus? Current diabetes reports 2, 216–222 (2002). 12643176 10.1007/s11892-002-0086-2

[b38] Choi C. S. . Paradoxical effects of increased expression of PGC-1alpha on muscle mitochondrial function and insulin-stimulated muscle glucose metabolism. Proc Natl Acad Sci USA 105, 19926–19931 (2008). 19066218 10.1073/pnas.0810339105 PMC2598730

[b39] Chao L. C. . Insulin Resistance and Altered Systemic Glucose Metabolism in Mice Lacking Nur77. Diabetes 58, 2788–2796 (2009). 19741162 10.2337/db09-0763 PMC2780886

[b40] Shan T. Z., Zhang P. P., Bi P. P. & Kuang S. H. Lkb1 Deletion Promotes Ectopic Lipid Accumulation in Muscle Progenitor Cells and Mature Muscles. J Cell Physiol 230, 1033–1041 (2015). 25251157 10.1002/jcp.24831 PMC4692267

[b41] Steinberg G. R. . Tumor necrosis factor alpha-induced skeletal muscle insulin resistance involves suppression of AMP-kinase signaling. Cell metabolism 4, 465–474 (2006). 17141630 10.1016/j.cmet.2006.11.005

[b42] Bandyopadhyay G. K., Yu J. G., Ofrecio J. & Olefsky J. M. Increased malonyl-CoA levels in muscle from obese and type 2 diabetic subjects lead to decreased fatty acid oxidation and increased lipogenesis; thiazolidinedione treatment reverses these defects. Diabetes 55, 2277–2285 (2006). 16873691 10.2337/db06-0062

[b43] Steinberg G. R. . Ciliary neurotrophic factor stimulates muscle glucose uptake by a PI3-kinase-dependent pathway that is impaired with obesity. Diabetes 58, 829–839 (2009). 19136654 10.2337/db08-0659 PMC2661597

[b44] Wilkes J. J., Nguyen M. T., Bandyopadhyay G. K., Nelson E. & Olefsky J. M. Topiramate treatment causes skeletal muscle insulin sensitization and increased Acrp30 secretion in high-fat-fed male Wistar rats. American journal of physiology. Endocrinology and metabolism 289, E1015–1022 (2005). 16030065 10.1152/ajpendo.00169.2005

[b45] Chen W. L., Chen Y. L., Chiang Y. M., Wang S. G. & Lee H. M. Fenofibrate lowers lipid accumulation in myotubes by modulating the PPARalpha/AMPK/FoxO1/ATGL pathway. Biochem Pharmacol 84, 522–531 (2012). 22687626 10.1016/j.bcp.2012.05.022

[b46] Liu X., Yuan H., Niu Y., Niu W. & Fu L. The role of AMPK/mTOR/S6K1 signaling axis in mediating the physiological process of exercise-induced insulin sensitization in skeletal muscle of C57BL/6 mice. Biochimica et biophysica acta 1822, 1716–1726 (2012). 22846606 10.1016/j.bbadis.2012.07.008

[b47] Ahmadian M. . Desnutrin/ATGL Is Regulated by AMPK and Is Required for a Brown Adipose Phenotype. Cell metabolism 13, 739–748 (2011). 21641555 10.1016/j.cmet.2011.05.002 PMC3148136

[b48] Chen J. P., Zhou X. H., Wu W. C., Wang X. X. & Wang Y. Z. FTO-dependent function of N6-methyladenosine is involved in the hepatoprotective effects of betaine on adolescent mice. J Physiol Biochem 71, 405–413 (2015). 26078098 10.1007/s13105-015-0420-1

[b49] Medina-Gomez G., Gray S. & Vidal-Puig A. Adipogenesis and lipotoxicity: role of peroxisome proliferator-activated receptor gamma (PPARgamma) and PPARgammacoactivator-1 (PGC1). Public health nutrition 10, 1132–1137 (2007). 17903321 10.1017/S1368980007000614

[b50] Wu Z. D. . Cross-regulation of C/EBP alpha and PPAR gamma controls the transcriptional pathway of adipogenesis and insulin sensitivity. Mol Cell 3, 151–158 (1999). 10078198 10.1016/s1097-2765(00)80306-8

[b51] Gerken T. . The obesity-associated FTO gene encodes a 2-oxoglutarate-dependent nucleic acid demethylase. Science 318, 1469–1472 (2007). 17991826 10.1126/science.1151710 PMC2668859

[b52] Bravard A. . FTO is increased in muscle during type 2 diabetes, and its overexpression in myotubes alters insulin signaling, enhances lipogenesis and ROS production, and induces mitochondrial dysfunction. Diabetes 60, 258–268 (2011). 20943749 10.2337/db10-0281 PMC3012179

[b53] Church C. . A mouse model for the metabolic effects of the human fat mass and obesity associated FTO gene. PLoS genetics 5, e1000599 (2009). 19680540 10.1371/journal.pgen.1000599 PMC2719869

[b54] Larder R., Cheung M. K., Tung Y. C., Yeo G. S. & Coll A. P. Where to go with FTO? Trends in endocrinology and metabolism: TEM 22, 53–59 (2011). 21131211 10.1016/j.tem.2010.11.001

[b55] Merkestein M. . FTO influences adipogenesis by regulating mitotic clonal expansion. Nat Commun 6, 6792 (2015). 25881961 10.1038/ncomms7792 PMC4410642

[b56] McMurray F. . Pharmacological inhibition of FTO. Plos One 10, e0121829 (2015). 25830347 10.1371/journal.pone.0121829 PMC4382163

[b57] Zhang J. L. . Expression and significance of fat mass and obesity associated gene and forkhead transcription factor O1 in non-alcoholic fatty liver disease. Chinese Med J-Peking 127, 3771–3776 (2014). 25382334

