# Supplementary material for: AMPK regulates lipid accumulation in skeletal muscle cells through FTO-dependent demethylation of N6-methyladenosine
Source: Sci Rep. 2017 Feb 8;7:41606. doi: 10.1038/srep41606 (PMC5296945; doi:10.1038/srep41606)
Supplement: Supplemental Data [file srep41606-s1.pdf]

AMPK regulates lipid accumulation in skeletal muscle cells through FTO-dependent demethylation of N^6-methyladenosine

Weiche Wu[1], Jie Feng[1], Denghu Jiang[1], Xihong Zhou[2], Qin Jiang[1], Min Cai[1], Xinxia Wang[1], Tizhong Shan[1*], Yizhen Wang[1*]

[1] College of Animal Science, Zhejiang University; Key Laboratory of Animal Nutrition & Feed Sciences, Ministry of Agriculture; Zhejiang Provincial Laboratory of Feed and Animal Nutrition; No. 866 Yuhangtang Road, Hangzhou, Zhejiang, 310058, P. R. China

[2] Institute of Subtropical Agriculture, The Chinese Academy of Science, Changsha, Hunan 410125, P. R. China

***Corresponding author:**

Email: tzshan@zju.edu.cn, yzwang321@zju.edu.cn

Fax:+86-0571-88982729

Tel.: +86-0571-88982729

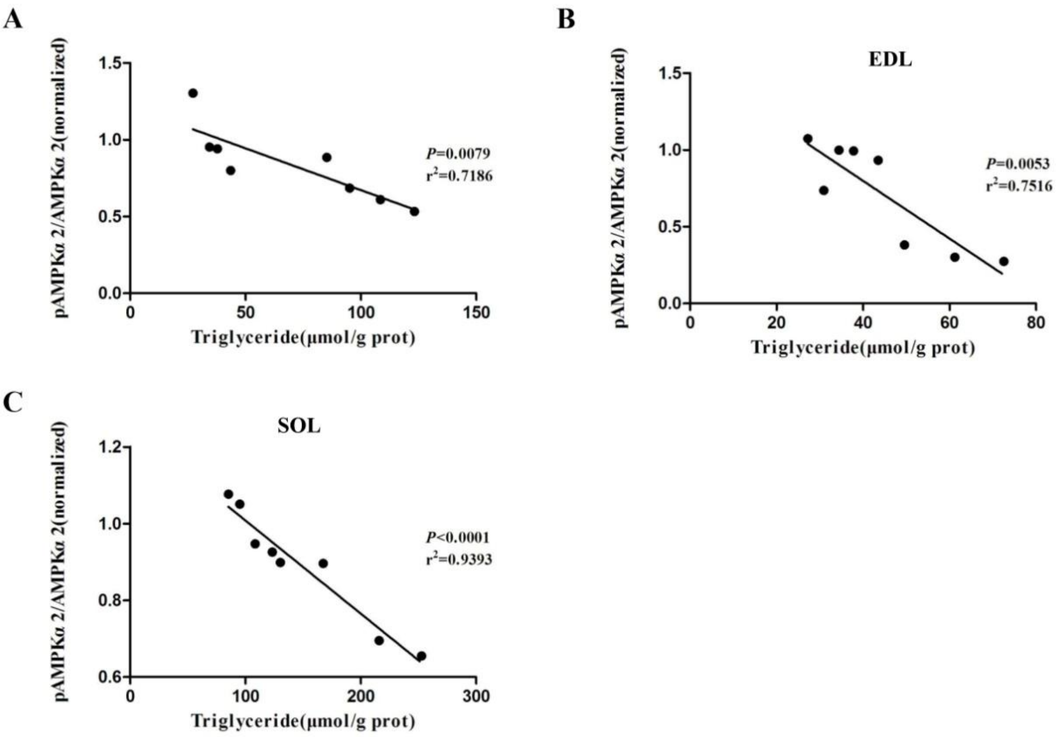

Supplemental Figure 1

Correlation between pAMPK levels and triglyceride content in muscle. (A) Correlation in the EDL and SOL of WT mice. (B, C) Correlation in the EDL (B) and SOL (C) of WT and OB mice. P value and R squared are presented in the graphs.

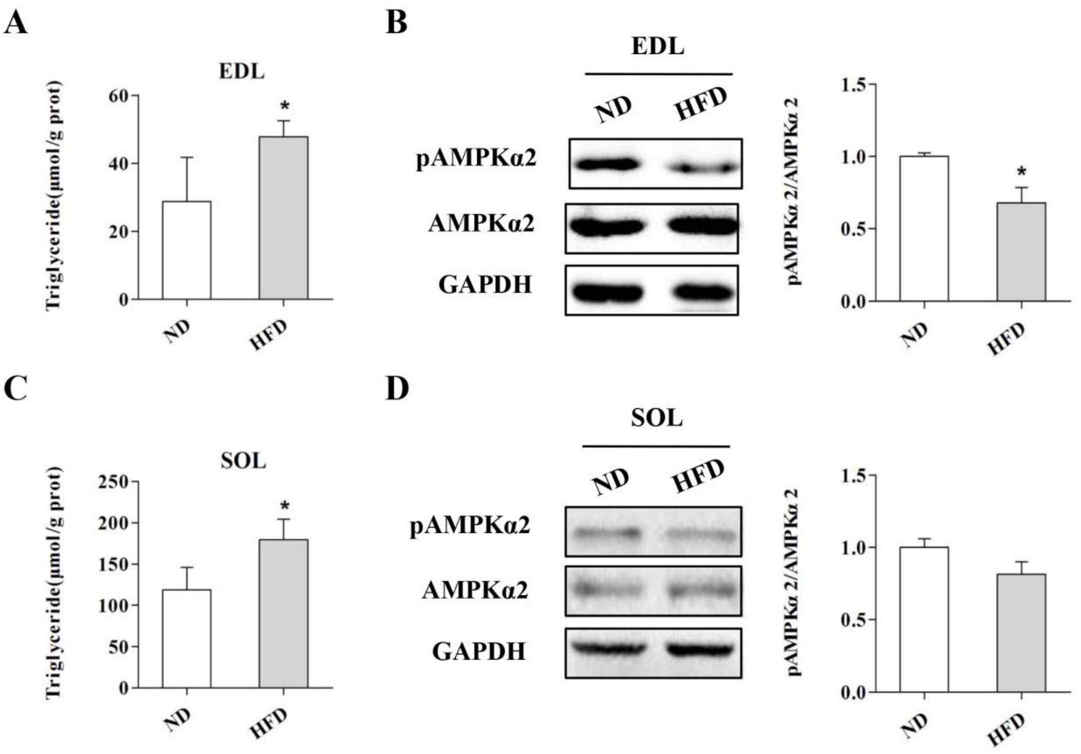

Supplemental Figure 2

The expression of AMPK in skeletal muscle is inversely correlated with lipid content.(A) The triglyceride content in the EDL of the HFD group and ND group.(B) The p-AMPKα2 levels in the EDL of the HFD group and ND group. (C) The triglyceride content in the SOL of the HFD group and the ND group.(D) The p-AMPKα2 levels in the SOL of the HFD group and ND group. The results are presented as the mean ± SEM. n=4-6. $*p < 0.05$.

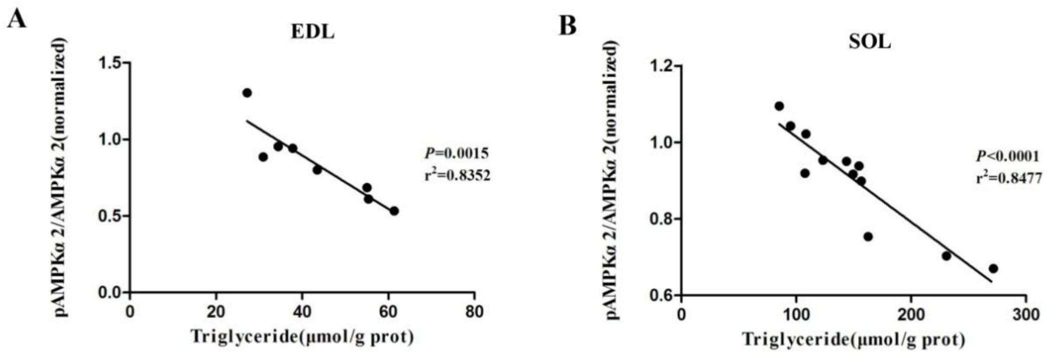

Supplemental Figure 3

Correlation analysis between pAMPK levels and triglyceride content in the EDL and SOL of ND- and HFD- fed mice. (A)Correlation in EDL. (B) Correlation in SOL. P value and R squared are presented in the graphs.

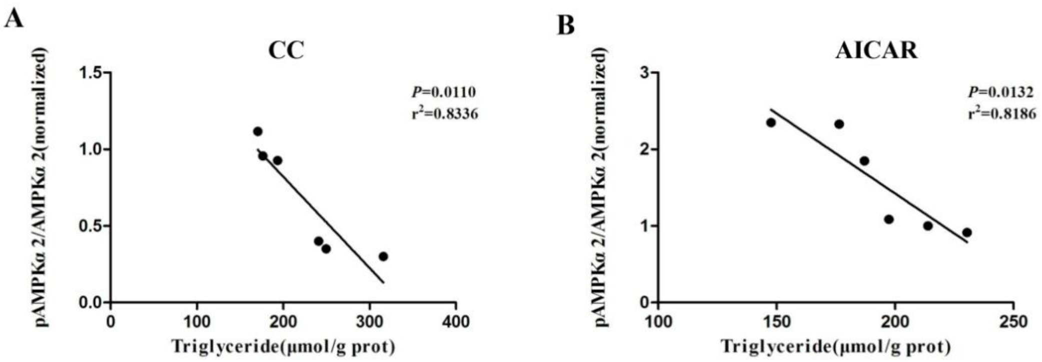

Supplemental Figure 4

Correlation between pAMPK levels and triglyceride content in C2C12 cells. (A)Correlation in the Vehicle and CC groups. (B) Correlation in the Vehicle and AICAR groups. P value and R squared are presented in the graphs.

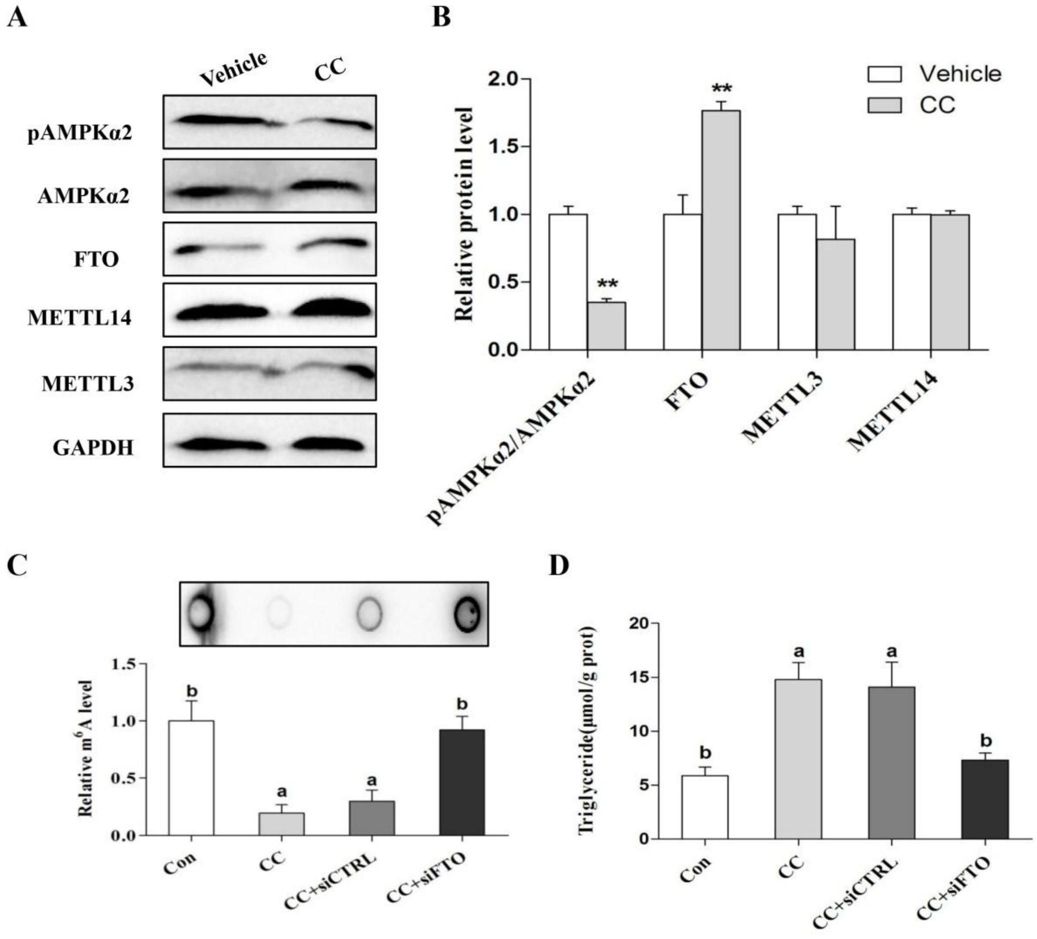

Supplemental Figure 5

Compound C upregulates FTO expression and decreases m^{6}A methylation in C2C12 cells.(A, B) The protein levels of p-AMPKα2, AMPKα2, FTO, METTL3 and METTL14 in C2C12 cells after CC treatment. (C, D) m^{6}A methylation (C) and the triglyceride content (D) after combined treatment with CC and siFTO. Data are presented as the mean ± SEM. n=3. *p< 0.05, ** p< 0.01.
